# Bioinformatics Identification of Lactate‐Associated Genes in Hepatocellular Carcinoma: G6PD's Role in Immune Modulation

**DOI:** 10.1002/cam4.70801

**Published:** 2025-03-21

**Authors:** Hao‐ran Qu, Chao‐qun Wang, Su‐juan Sun, Wen‐wen Zhang, Cheng‐hao Liu, Xuan‐shuang Du, Yao‐yi‐ao Shu, Xi‐cheng Wang, Qin Pan, Feng‐ling Luo, Hong‐yan Wu, Xiao‐lian Zhang, Min Liu

**Affiliations:** ^1^ State Key Laboratory of Virology, Hubei Province Key Laboratory of Allergy and Immunology, Department of Immunology, Taikang Medical School (School of Basic Medical Sciences) Wuhan University Wuhan China; ^2^ Frontier Science Center for Immunology and Metabolism, Department of Allergy Zhongnan Hospital Wuhan University School of Medicine Wuhan China; ^3^ Department of Neurosurgery Renmin Hospital of Wuhan University Wuhan China; ^4^ Central Laboratory Renmin Hospital of Wuhan University Wuhan China; ^5^ Hubei Key Laboratory of Tumor Microenvironment and Immunotherapy China Three Gorges University Yichang China; ^6^ School of Basic Medicine China Three Gorges University Yichang China

**Keywords:** G6PD, hepatocellular carcinoma, lactate metabolism, PD‐L1, prognostic biomarkers

## Abstract

**Background:**

Hepatocellular carcinoma (HCC) is a major global health issue, with poor prognosis often associated with dysregulated metabolic pathways, especially lactate metabolism. This study explored the prognostic significance of lactate‐associated genes in HCC and their potential as therapeutic targets.

**Methods:**

We analyzed RNA‐seq and clinical data from 374 patients with HCC from The Cancer Genome Atlas (TCGA) database. Using Cox regression, LASSO analysis, and Kaplan–Meier survival curves, we identified key lactate‐associated genes associated with patient outcomes. Functional validations, including Western blot, flow cytometry, and molecular docking studies, were performed to confirm the biological impact of these genes.

**Results:**

G6PD, IK, and CALML5 were identified as significant prognostic markers for HCC. A prognostic model was developed that effectively stratified patients into risk groups, which correlated with survival. G6PD's role in immune modulation and its potential as a drug target were validated through biochemical assays and computational analyses. Functional assays in HepG2 cells confirmed that alterations in G6PD expression affect T cell activity, with knockdown enhancing IFN‐γ production and overexpression inhibiting it, demonstrating G6PD's role in immune evasion.

**Conclusions:**

This study establishes lactate metabolism genes, particularly G6PD, as key prognostic markers in HCC. The validation of G6PD's immunomodulatory effects further supports its potential as a therapeutic target for strategies aimed at enhancing immune surveillance and treatment outcomes in HCC.

## 1 Introduction

Hepatocellular carcinoma (HCC) is a leading cause of cancer‐related deaths globally, characterized by its aggressive nature and poor prognosis [1]. The metabolic reprogramming of cancer cells, including altered lactate metabolism, plays a crucial role in HCC progression and therapeutic resistance. Lactate, traditionally viewed as a by‐product of anaerobic glycolysis, has emerged as a key molecule in cancer, influencing both the tumor microenvironment and cancer cell signaling [2, 3, 4].

Recent studies have linked lactate metabolism to key pathways involved in cancer proliferation, metastasis, and immune evasion [5]. The Warburg effect [6], characterized by increased glucose uptake and lactate production even in the presence of oxygen, highlights the metabolic shift in cancer cells favoring lactate production. This metabolic shift is not merely a survival mechanism but also a strategic alteration that supports invasiveness and therapy resistance [7, 8].

Lactate's role extends beyond its metabolic functions; it serves as a signaling molecule that alters gene expression, modifies enzyme activities, and influences immune cell behavior in the tumor microenvironment [9]. Lactate inhibits immune surveillance by repressing the activity of immune cells, including T cells and natural killer cells, thereby contributing to immune evasion mechanisms in tumors [10, 11, 12].

Given the profound impact of lactate on HCC progression, identifying genetic markers linked to lactate metabolism may provide new prognostic tools and therapeutic targets. Studies have shown that genes involved in lactate production and transport are overexpressed in HCC and are correlated with poor patient outcomes [13, 14, 15]. Exploring the genetic underpinnings of lactate metabolism in HCC could provide valuable insights into its pathogenesis and uncover new avenues for targeted therapy.

This study aimed to identify and evaluate the prognostic significance of lactate‐associated genes in HCC by using bioinformatics approaches. We focused specifically on G6PD because of its strong association with tumor progression and immune cell infiltration, as revealed in large‐scale transcriptomic datasets. We also investigated its role in regulating immune checkpoints, particularly PD‐L1, through the mTOR pathway, and explored its potential as a therapeutic target to enhance anti‐tumor immunity in HCC. By integrating single‐cell RNA sequencing (scRNA‐seq) data, the Cancer Genome Atlas (TCGA) datasets, and functional assays, this study aims to provide new insights into G6PD's role in lactate metabolism, immune modulation, and HCC prognosis. Our findings highlight the complex interplay between metabolic reprogramming and immune regulation, providing new perspectives on how metabolic enzymes like G6PD contribute to tumor immune evasion and therapeutic resistance.

## 2 Results

### 2.1 Identification and Prognostic Relevance of Lactate‐Associated Genes

**FIGURE 1:**
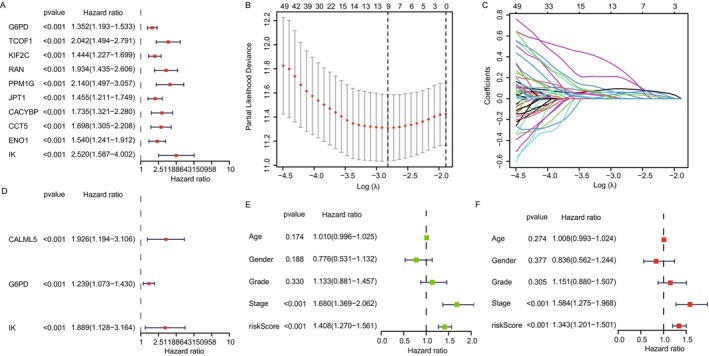
Evaluation and validation of lactate metabolism‐related genes as prognostic markers in HCC. (A) Univariate Cox regression analysis showing hazard ratios for the top 10 lactate metabolism‐related genes significantly associated with HCC prognosis, emphasizing their potential as biomarkers. (B) Visualization of the optimal lambda (λ) selection in Lasso regression, which minimizes model deviance and is used to identify the most significant genes for further analysis. (C) Coefficient profiles of selected genes across different lambda values in Lasso regression, illustrating the regularization path and the stability of each gene's coefficient. (D) Multivariate Cox regression analysis demonstrating the prognostic impact of the final selected genes—CALML5, G6PD, and IK—each contributing uniquely to the risk model. (E and F) Prognostic models incorporating clinical variables and gene‐based risk scores, illustrating the significant variables in both univariate and multivariate analyses that influence patient survival and categorize patients into high‐ and low‐risk groups based on their median risk scores.

We used data from 371 hepatocellular carcinoma (HCC) patients with complete survival information from The Cancer Genome Atlas (TCGA) to evaluate the prognostic significance of lactate‐associated genes. Univariate Cox regression analysis initially identified 128 lactate‐associated genes significantly correlated with HCC prognosis from a list of 332 genes linked to lactate processes according to the literature [16]. The top 10 genes significantly associated with HCC prognosis were G6PD, TCOF1, KIF2C, RAN, PPM1G, JPT1, CACYBP, CCT5, ENO1, and IK (Figure 1A). LASSO regression was then used to refine the selection of candidate prognostic genes, identifying nine potential genes from the list (Figure 1B,C). To confirm the independent prognostic value of the selected genes, we performed multivariate Cox regression analysis. This analysis identified three critical prognostic genes: CALML5, G6PD, and IK (Figure 1D). These genes were the strongest predictors of overall survival in HCC patients, adjusting for other clinical variables such as age, gender, and cancer stage. A prognostic model was constructed using these three genes (CALML5, G6PD, and IK), and risk scores were calculated based on their gene expression levels. Patients were stratified into high‐ and low‐risk groups based on median risk scores (Figure 1E). The high‐risk group showed significantly worse overall survival than the low‐risk group, highlighting the prognostic relevance of the lactate‐associated gene model. Finally, we performed univariate and multivariate Cox regression analyses to assess the impact of clinical variables, such as gene score and tumor stage, on prognosis. Both analyses showed that the gene score from the prognostic model and tumor stage were significantly correlated with survival outcomes in HCC patients (Figure 1F). Our analysis identified G6PD, CALML5, and IK as critical prognostic markers for HCC based on their association with lactate metabolism and survival. The prognostic model, incorporating these three genes, effectively stratifies patients into high‐ and low‐risk groups, offering valuable insights for personalized treatment strategies in HCC.

### 2.2 Construction and Validation of Lactate‐Associated Gene Prognostic Model

**FIGURE 2:**
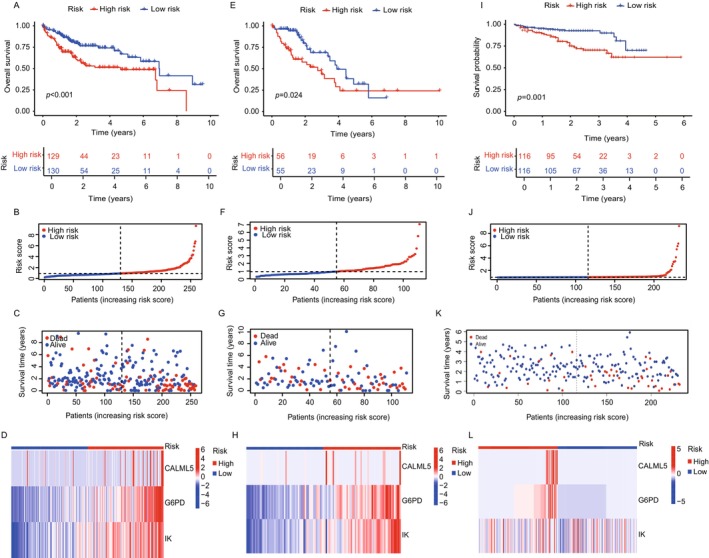
Construction and validation of lactate‐associated gene prognostic model in HCC. (A), (E), and (I) Kaplan–Meier survival curves for the training set, internal validation set, and external validation set, respectively, demonstrating the overall survival differences between high‐ and low‐risk groups as defined by the prognostic model, with p‐values indicating significant separation. (B), (F), and (J) Risk score distributions for the training set, internal validation set, and external validation set, respectively, highlighting the progression from low to high risk across the patient spectrum. (C), (G), and (K) Survival status dot plots of patients in the training set, internal validation set, and external validation set, respectively, plotting survival time against the risk score, with markers indicating deceased (red) and alive (blue) status. (D), (H), and (L) Heatmaps of gene expression profiling in the training set, internal validation set, and external validation set, illustrating the expression levels of key prognostic genes across risk groups, with red indicating high expression and blue indicating low expression.

We constructed and validated a prognostic model based on lactate‐associated genes (CALML5, G6PD, and IK), using the TCGA LIHC cohort, divided into training (Figure 2A–D) and internal validation (Figure 2E–H) sets, along with the external validation cohort (LIHC‐JP) (Figure 2I–L). The model aimed to assess the predictive value of lactate metabolism‐related genes in HCC and was evaluated across different datasets. In the training set, patients were stratified into high‐ and low‐risk groups based on risk scores derived from the lactate‐associated gene expression model. Kaplan–Meier survival analysis revealed a significant survival difference between the two groups. Patients in the high‐risk group showed significantly poorer survival than those in the low‐risk group, with increased mortality and decreased overall survival (OS) as risk scores increased (Figure 2A,E). The distribution of risk scores and survival status was visualized to highlight survival differences (Figure 2B,C,F,G). A heatmap of the three key genes in the prognostic model was generated to compare their expression patterns between high‐ and low‐risk groups (Figure 2D,H). This analysis showed that the expression of these genes was significantly higher in the high‐risk group than in the low‐risk group, reinforcing the association between elevated gene expression and poor prognosis. The heatmap also illustrated the distinct molecular characteristics of each risk group, confirming the prognostic relevance of lactate‐associated genes in HCC. The external validation cohort (LIHC‐JP) was used to validate the predictive value of the lactate‐associated gene model. Similar to the training set, the high‐risk group in the validation set showed significantly worse prognosis than the low‐risk group (Figure 2I), confirming the robustness of the model across datasets. The external validation heatmap (Figure 2L) showed consistent expression patterns of the three key genes, confirming their relevance in predicting patient outcomes in an independent cohort. The distribution of risk scores and survival analysis in the external validation cohort mirrored the findings in the training set, further validating the prognostic utility of the model (Figure 2J,K). These findings suggest that lactate metabolism‐related genes may serve as valuable biomarkers for prognosis in HCC.

### 2.3 Independent Prognostic Analysis and PCA

**FIGURE 3:**
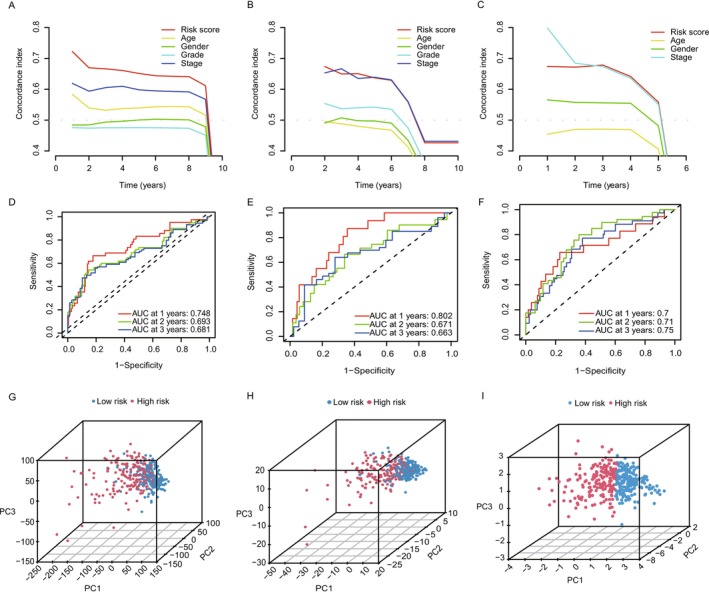
Independent prognostic analysis and PCA of lactate‐associated genes. (A‐C) Concordance index plots illustrating the independent prognostic value of risk score, age, gender, grade, and stage over 1–10 years, demonstrating the model's ability to predict overall survival across time. (D‐F) Receiver operating characteristic (ROC) curves for 1‐, 2‐, and 3‐year overall survival predictions across the entire cohort, highlighting the area under the curve (AUC) values to evaluate the prognostic model's accuracy. (G‐I) Principal component analysis (PCA) plots showing the distribution of patient samples in the TCGA dataset based on their risk scores, distinguishing between high‐risk (red) and low‐risk (blue) groups, visually validating the model's effectiveness in stratifying patients by genetic profiles.

We performed Decision Curve Analysis (DCA) to evaluate the clinical utility of our lactate‐associated gene prognostic model. DCA plots (Figure 3A–C) showed that the model provides significant net clinical benefits across a wide range of threshold probabilities. This indicates that the model offers valuable predictive information for clinicians, assisting in decision‐making for overall survival (OS) predictions. The net benefit curve suggests that our model outperforms traditional strategies, including clinical variables alone, by identifying patients who would benefit most from early intervention based on risk stratification. The model's predictive accuracy was further assessed using Receiver Operating Characteristic (ROC) curves for 1‐, 2‐, and 3‐year overall survival (OS) predictions. As shown in Figure 3D–F, the Area Under the Curve (AUC) values for both the TCGA cohort and external validation cohort were consistently above 0.65, with some exceeding 0.7 and even reaching 0.8 for certain time points. These findings highlight the strong performance of our model in accurately predicting OS, further supporting its potential utility in clinical practice for long‐term survival predictions in HCC patients. To further validate the model's ability to stratify patient risk, we performed Principal Component Analysis (PCA) on the full TCGA dataset, focusing on lactate‐associated genes and those identified in the risk model. PCA results, shown in Figure 3G–I, revealed clear separation between high‐risk and low‐risk groups based on gene expression profiles. The PCA plots show that the risk genes effectively distinguish patients with better prognosis (low‐risk) from those with worse prognosis (high‐risk), supporting their utility in risk stratification. Our analysis confirms that the lactate‐associated gene prognostic model is a robust and clinically relevant tool for predicting overall survival in HCC patients.

### 2.4 Enrichment Analysis of High‐ and Low‐Risk Groups in HCC

**FIGURE 4:**
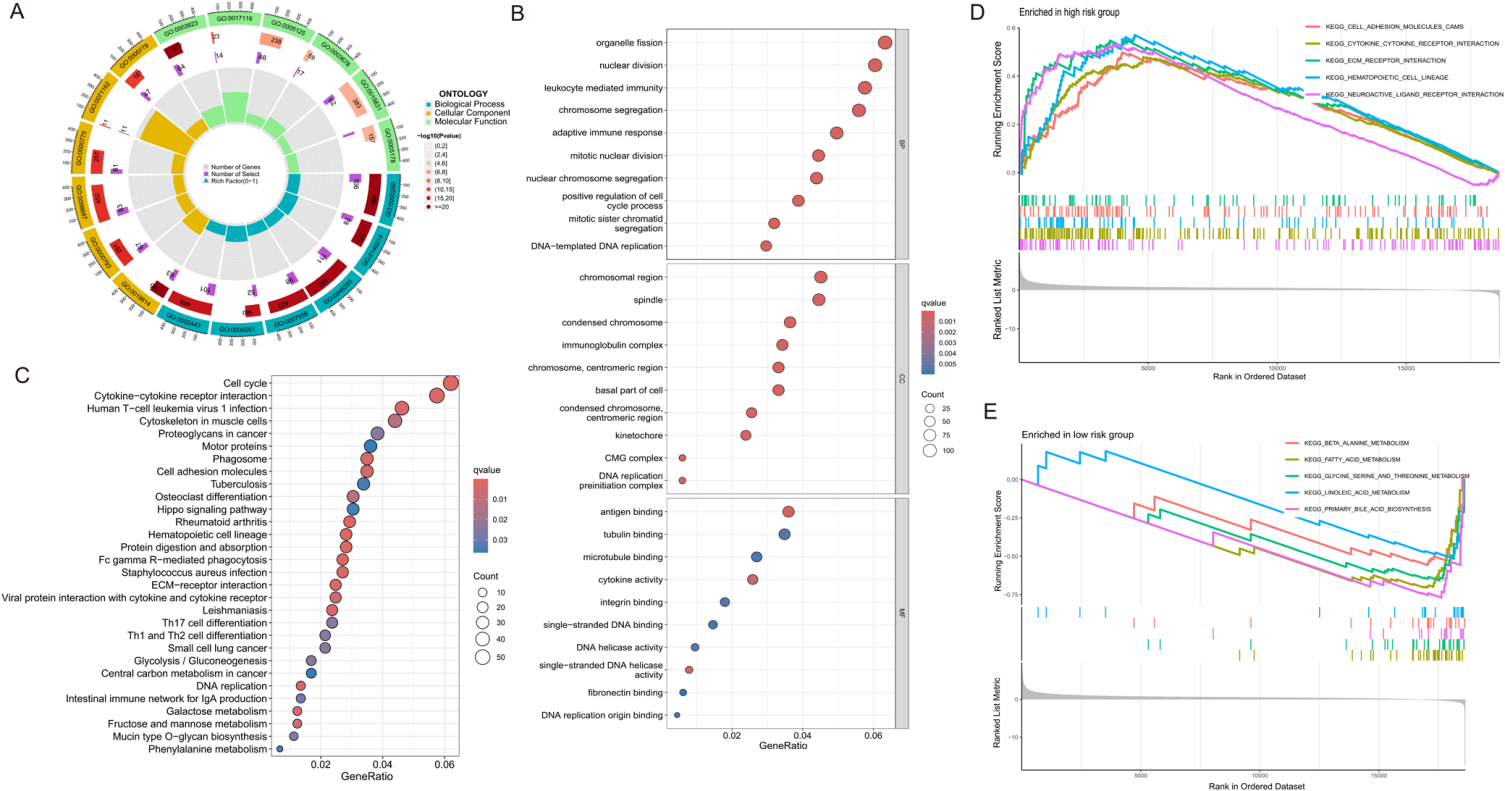
Enrichment analysis of lactate‐associated genes. (A) Circular representation of gene ontology (GO) enrichment across three domains—biological process, cellular component, and molecular function—with inner rings depicting specific GO terms and outer rings showing significant genes in high‐ and low‐risk groups. (B) Dot plot illustrating the top enriched Biological Process terms, ranked by *q*‐value, emphasizing processes like the cell cycle, cytokine–cytokine receptor interaction, and glycolysis, which are prominent in high‐risk groups. (C) Bubble plot detailing the KEGG pathway analysis identifying critical pathways that differentiate between high‐ and low‐risk groups, such as p53 signaling and cell adhesion molecules, with circle sizes representing the gene ratio and color intensity reflecting the *q*‐value. (D) GSEA enrichment curves for high‐risk groups showing the most significantly enriched pathways, such as KEGG_CELL_ADHESION_MOLECULES_CAMs, with colors representing different pathways. (E) GSEA enrichment curves for low‐risk groups, highlighting pathways like KEGG_ALANINE_METABOLISM and KEGG_FATTY_ACID_METABOLISM, suggesting a distinct metabolic profile associated with lower cancer risk.

The circular plot illustrates gene ontology (GO) categories, highlighting key biological processes, such as cell cycle regulation and immune responses, which are more active in the high‐risk group. This visualizes significant gene expression differences related to patient prognosis (Figure 4A). A dot plot details the top enriched Biological Processes, highlighting critical activities such as nuclear division and chromosome segregation, suggesting increased cellular proliferation in high‐risk patients (Figure 4B). The bubble plot illustrates KEGG pathway analysis, identifying critical pathways, such as the cell cycle and cytokine–cytokine receptor interaction, associated with high‐risk groups (Figure 4C). GSEA plots compare high‐risk and low‐risk groups, with high‐risk patients showing enrichment in pathways related to cell adhesion and metastasis, while low‐risk patients exhibit activity in metabolic pathways, potentially correlating with better outcomes (Figure 4D,E).

### 2.5 Immune Infiltration and Checkpoint Analysis

**FIGURE 5:**
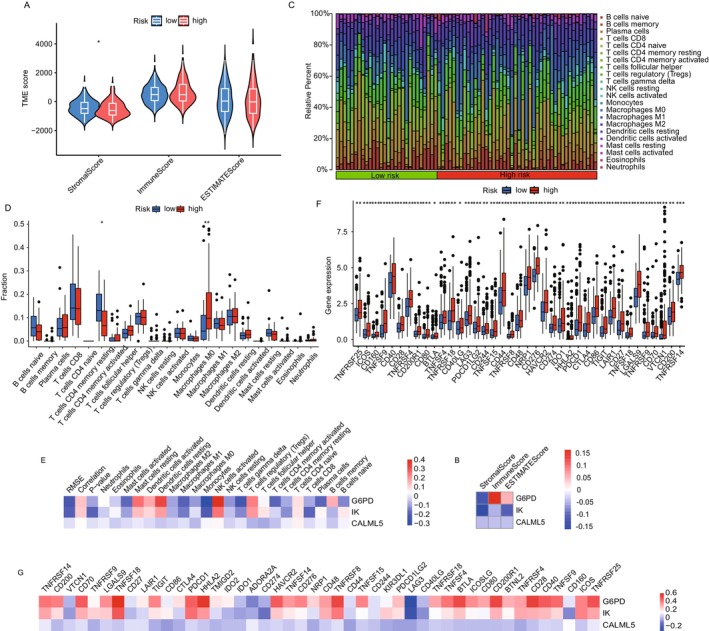
Comprehensive immune profile and checkpoint expression in high‐ and low‐risk hepatocellular carcinoma groups. (A) Violin plots depicting the distribution of ESTIMATE scores, highlighting differences in immune and stromal scores between high‐ and low‐risk groups. (B) Heatmap showing the correlation between key lactate‐associated genes (G6PD, IK, and CALML5) and immune scores, highlighting the impact of these genes on the tumor microenvironment. (C) Bar plots illustrating the relative proportions of 22 immune cell types, assessed by single‐sample gene set enrichment analysis (ssGSEA), across high‐ and low‐risk groups. (D) Box plots highlighting the variability and differential abundance of specific immune cells between the risk groups. (E) Heatmap summarizing the correlation coefficients between the expression of G6PD, IK, and CALML5 genes and the infiltration levels of various immune cells. (F) Bar graph depicting gene expression levels of major immune checkpoints in high‐ and low‐risk groups. (G) Correlation heatmap displaying the relationships between immune checkpoint expression and lactate‐associated genes.

The tumor microenvironment (TME) plays a crucial role in tumor progression and response to immunotherapy. To explore the relationship between immune cell infiltration and lactate‐associated gene expression, we analyzed the distribution of immune cells and immune checkpoint expression in high‐risk and low‐risk groups based on the lactate‐associated gene model. Our analysis revealed that the low‐risk group exhibited a significantly higher proportion of stromal cells, suggesting a more active stromal component in these patients (Figure 5A). This finding suggests that stromal cells in the tumor microenvironment may contribute to more favorable immune conditions in low‐risk patients. We also observed a strong positive correlation between G6PD expression and immune scores (Figure 5B). This suggests that higher G6PD expression may shape the immune landscape, potentially promoting immune cell infiltration and influencing the immune response in HCC. This positive correlation with immune scores highlights the potential role of G6PD in modulating the TME, especially in immune activation. To further assess immune cell infiltration, we used single‐sample gene set enrichment analysis (ssGSEA) to evaluate the levels of 22 immune cell types in the high‐ and low‐risk groups. Our results indicated increased macrophage (M0) infiltration in high‐risk groups, whereas CD4 memory resting cells were more prevalent in low‐risk groups (Figure 5C–E). This suggests that high‐risk tumors may exhibit a more immunosuppressive TME, with macrophages, often involved in promoting immune evasion, being more abundant in these patients. Conversely, the increased presence of CD4 memory resting cells in the low‐risk group may reflect a more balanced immune environment, potentially enhancing immune surveillance and tumor control. We further analyzed the expression of key immune checkpoints, including CTLA‐4, CD274 (PD‐L1), PDCD1 (PD‐1), LAG3, and HAVCR2. These checkpoints play a crucial role in immune evasion and tumor progression. Our findings revealed differential expression of these checkpoints between the high‐ and low‐risk groups (Figure 5F,G). Notably, G6PD and IK expression levels were strongly correlated with immune checkpoint expression, especially in the high‐risk group. This suggests that G6PD and IK may regulate immune evasion by modulating immune checkpoint pathways, further supporting their potential role in promoting immune suppression and escape in HCC.

### 2.6 Mutation Analysis and Drug Sensitivity in HCC Risk Groups

**FIGURE 6:**
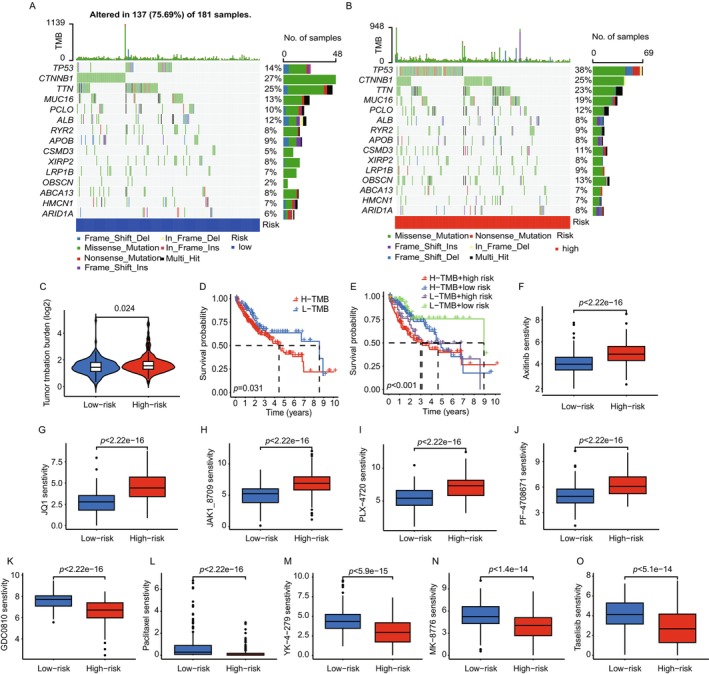
Mutation analysis and drug sensitivity correlation with risk groups in HCC. (A‐B) Mutation landscapes depicting the frequency and types of somatic mutations in high‐risk and low‐risk groups, highlighting key genes with significant differences. (C) Violin plots illustrating the distribution of tumor mutational burden (TMB) in high‐ and low‐risk groups, with a higher TMB observed in the high‐risk group. (D‐E) Survival curves comparing overall survival based on TMB and risk categories, emphasizing the prognostic significance of TMB alongside risk scores. (F‐O) Box plots presenting the differential sensitivity of high‐ and low‐risk groups to various chemotherapy agents, as indicated by half‐maximal inhibitory concentration (IC50) values, demonstrating distinct drug responses between the groups. Each plot corresponds to a specific therapeutic agent, suggesting potential for tailored treatment based on risk stratification.

Somatic mutation analysis within the TCGA dataset revealed distinct mutational profiles between high‐risk and low‐risk groups (Figure 6A,B). High‐risk patients exhibited a higher frequency of mutations in key oncogenes and tumor suppressor genes, such as TP53 and CTNNB1. Notably, high‐risk patients showed a higher tumor mutational burden (TMB), suggesting a potential correlation with poor prognosis. Tumor Mutational Burden analysis (Figure 6C) quantified TMB, revealing that high‐risk patients consistently exhibited higher TMB values compared to low‐risk groups. This difference was statistically significant and supports the association between higher TMB and increased risk scores. Survival analysis showed that patients with higher TMB, particularly those in the high‐risk group, had poorer overall survival rates (Figure 6D,E). These findings suggest that TMB could serve as an independent prognostic factor in risk stratification. Drug sensitivity testing using data from the GDSC database revealed variable responses to chemotherapy agents between the groups. High‐risk patients generally exhibited reduced sensitivity to drugs like sorafenib and doxorubicin, as reflected by higher IC50 values. Conversely, some agents, such as cisplatin and paclitaxel, exhibited relatively lower IC50 values in high‐risk groups, suggesting a nuanced response pattern that may inform therapeutic decisions (Figure 6F–O).

### 2.7 Gene Coexpression Network Analysis

**FIGURE 7:**
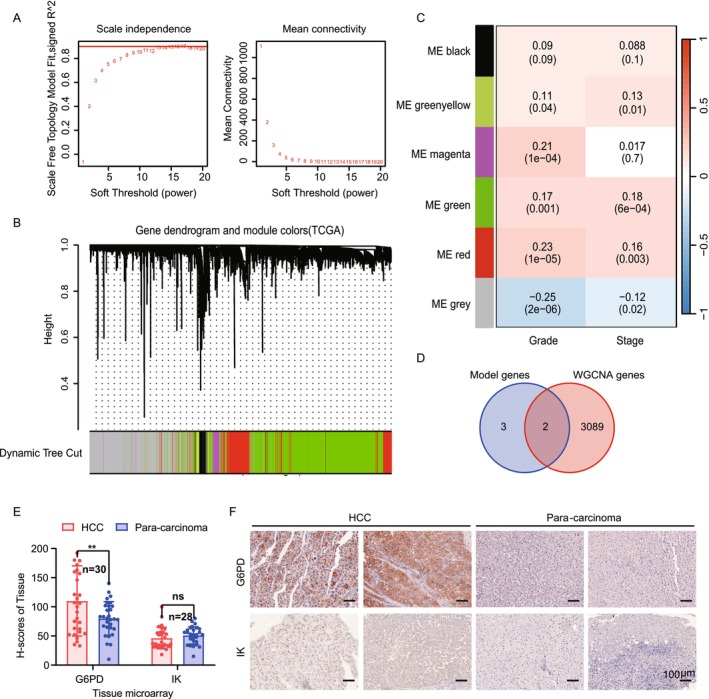
Gene coexpression network analysis and correlation with hepatocellular carcinoma progression. (A) Scale independence plot for different soft‐thresholding powers, indicating the optimal power where the scale‐free topology fit index reaches 0.9. (B) Gene dendrogram obtained through average linkage hierarchical clustering, with module colors assigned at the dynamic tree cut to identify gene co‐expression modules in the TCGA hepatocellular carcinoma dataset. (C) Heatmap showing the correlation between gene modules and clinical traits of HCC, including tumor grade and stage. Color intensity represents correlation coefficients, with red indicating positive and blue indicating negative correlations. (D) Venn diagram illustrating the overlap between genes identified in the predictive model and those in the significant “Red” and “Green” WGCNA modules. (E‐F) Immunohistochemical staining for G6PD and IK was performed on HCC tumor microarray and statistically compared the expression of these two genes in carcinoma and para‐carcinoma by using H‐scores. (E) Statistical chart. (F) Representative images. Scale bar: 100 μm.

Using weighted gene co‐expression network analysis (WGCNA), we identified gene modules linked to tumor characteristics in HCC. The optimal soft‐thresholding power for constructing a scale‐free network was *β* = 13, with a scale‐free fit index of 0.90, indicating a robust network topology (Figure 7A). Hierarchical clustering of genes revealed six distinct gene modules, each represented by a unique color (Figure 7B). The “Red” and “Green” modules showed significant correlations with clinical traits. The “Red” module had a strong positive correlation with tumor grade, indicating its association with more aggressive tumor features. In contrast, the “Green” module was most strongly correlated with tumor stage, suggesting its role in HCC progression (Figure 7C). Further analysis with Venn diagrams identified two genes, G6PD and IK, overlapping between our prognostic model's lactate‐associated genes and the “Red” and “Green” modules, highlighting their relevance in HCC pathology (Figure 7D). Based on stepwise Cox regression analysis and LASSO regression, which identified G6PD and IK as the most robust independent prognostic markers, while CALML5 exhibited weaker prognostic significance (*p* > 0.05 in multivariate analysis). To enhance the model's predictive accuracy and clinical applicability, we refined it to focus on the two strongest predictors. Next, we performed immunohistochemical (IHC) analysis on the HCC tissue microarray (TMA), and H‐score quantification was used to assess protein expression levels (Figure 7E,F). The analysis revealed that G6PD was significantly overexpressed in HCC tissues compared to adjacent normal tissues, suggesting its potential role in HCC progression. However, there was no significant difference in IK, which may be related to the quality of the antibody and the small number of samples. Furthermore, IHC staining data from The Human Protein Atlas database (https://www.proteinatlas.org) corroborated these findings, showing elevated expression of G6PD and IK in HCC tissues relative to normal liver tissues (Figure S1). This consistency across datasets strengthens the evidence supporting G6PD and IK as potential biomarkers for tumor progression and promising therapeutic targets.

### 2.8 Single‐Cell Transcriptomic Analysis

**FIGURE 8:**
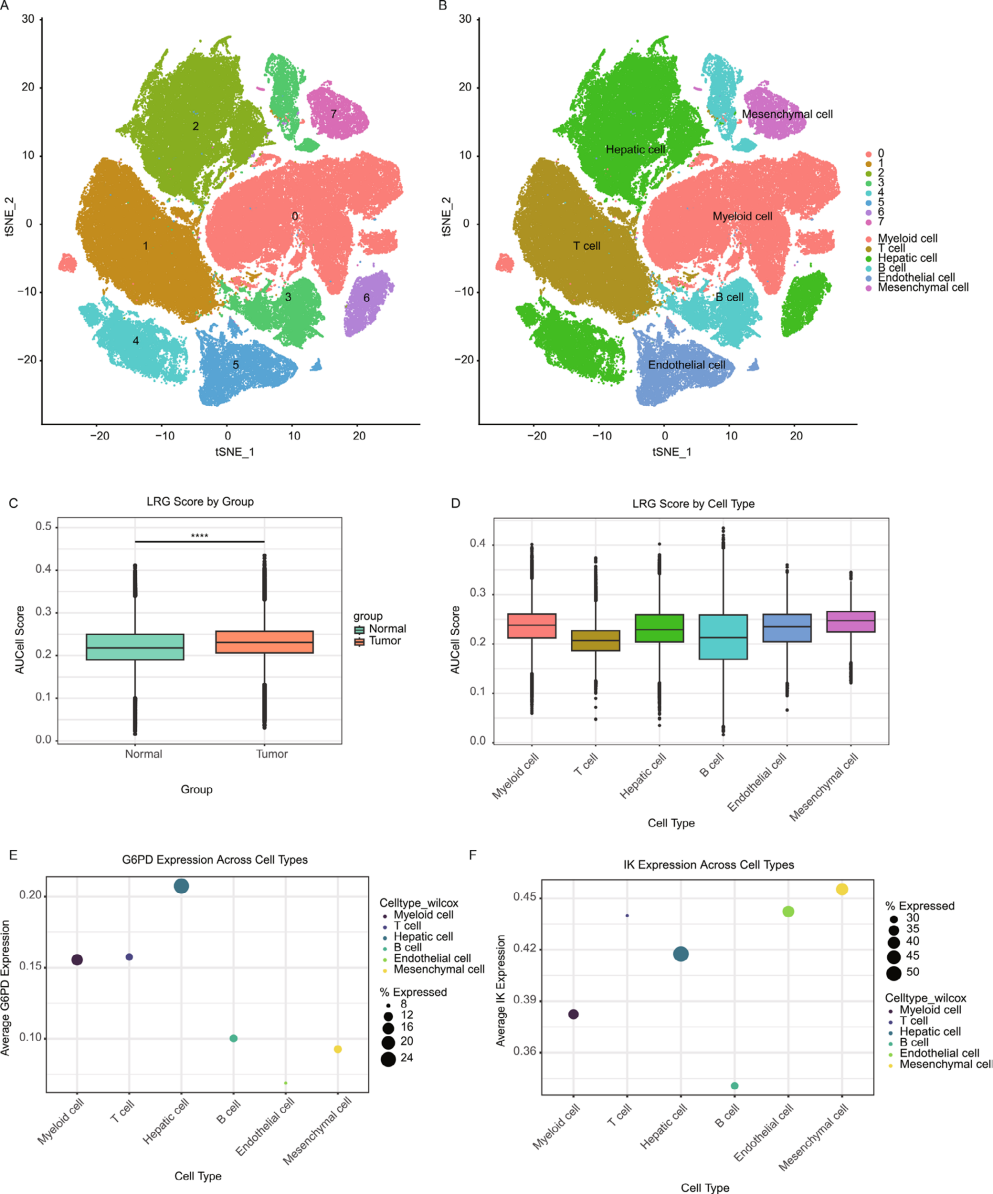
Single‐cell transcriptomic analysis of lactate‐associated genes in HCC. (A) t‐SNE plot illustrating the clustering of single cells from HCC and normal liver tissues, with clusters annotated according to their corresponding cell types. (B) t‐SNE plot displaying the distribution of tumor and normal cells across the clusters. (C) Boxplot comparing lactate‐related gene (LRG) scores between normal and tumor groups, showing significantly higher scores in tumor cells. (D) Boxplot displaying the distribution of LRG scores across different cell types, indicating higher scores in mesenchymal and myeloid cells. (E) Dot plot showing the average expression of G6PD across various cell types, with higher expression levels observed in hepatic and mesenchymal cells. (F) Dot plot showing the average expression of IK across cell types, with notable enrichment in myeloid and mesenchymal cells.

We analyzed single‐cell RNA sequencing (scRNA‐seq) data to explore the cellular heterogeneity and expression profiles of lactate‐associated genes in HCC. Using t‐SNE dimensionality reduction, cells were grouped into distinct clusters representing major cell types, including T cells, hepatic cells, endothelial cells, myeloid cells, and mesenchymal cells (Figure 8A,B). Analysis of lactate‐related gene (LRG) scores, based on key lactate‐associated gene expression, showed significantly higher scores in tumor cells compared to normal cells (Figure 8C). Stratification by cell type revealed that mesenchymal and myeloid cells had the highest LRG scores, indicating their critical role in lactate metabolism within the tumor microenvironment (Figure 8D). Further investigation of specific lactate‐associated genes revealed distinct expression patterns across cell types. G6PD, a key gene in lactate metabolism, was predominantly expressed in hepatic and mesenchymal cells (Figure 8E). Similarly, IK expression was enriched in myeloid and mesenchymal cells, highlighting its potential role in the metabolic reprogramming of these cell populations (Figure 8F). These findings highlight the significance of lactate‐associated genes in driving metabolic alterations and tumor progression at the single‐cell level. Given G6PD's prominent role in lactate metabolism and its expression in key cell types linked to tumor progression, we selected G6PD for further functional validation.

### 2.9 Validation of G6PD Expression in HCC

To explore the functional impact of G6PD on immune responses in HCC, we performed functional assays. The TCGA LIHC dataset revealed that G6PD is significantly overexpressed in tumor tissues compared to normal tissues (Figure S2A). Kaplan–Meier survival analysis indicated that high G6PD expression is associated with poor prognosis in HCC patients (HR = 2.15, log rank *p* = 8.6e‐05; Figure S2B). Stratification by cancer stage revealed a progressive increase in G6PD expression with advancing stages (Figure S2C), and nodal metastasis (N1) correlated with higher G6PD levels compared to nonmetastatic cases (Figure S2D). Age‐specific analysis further revealed that older patients had significantly higher G6PD expression than younger patients (Figure S2E). These findings underscore the association of G6PD with tumor progression and adverse clinical outcomes in HCC. Further analysis in liver cancer cell lines (HepG2, Huh7, and MHCC97H) revealed significantly higher G6PD mRNA and protein expression in cancer cells compared to the normal liver cell line L02 by western blot assays (Figure S2F–G), suggesting that G6PD overexpression contributes to the metabolic alterations characteristic of HCC cells.

### 2.10 G6PD and Immune Modulation in HCC

Our analysis of G6PD in HCC indicates its significant role in regulating immune responses, particularly in CD8^+^ T cell activation. Immune infiltration analysis (Figure S2) showed that G6PD expression positively correlated with CD8^+^ T cell infiltration in the tumor microenvironment (Figure S2A–D). G6PD expression was also associated with immune cell types involved in immune suppression, such as macrophages. This suggests that G6PD may modulate both immune activation and suppression within the tumor. Functional assays showed that G6PD knockdown in HepG2 cells enhanced CD8^+^ T cell proliferation and IFN‐γ secretion, indicating increased T cell activation (Figure S3E). In contrast, G6PD overexpression suppressed T cell activation, supporting the hypothesis that G6PD may contribute to immune evasion. These results suggest that G6PD modulates the immune response by regulating the tumor's ability to influence CD8^+^ T cell activity. The effects of G6PD on immune cell activation and suppression markers, such as CD274 (PD‐L1) in Figure 5, highlight its potential role in immune checkpoint regulation.

### 2.11 G6PD Regulates PD‐L1 Expression via the mTOR Pathway

**FIGURE 9:**
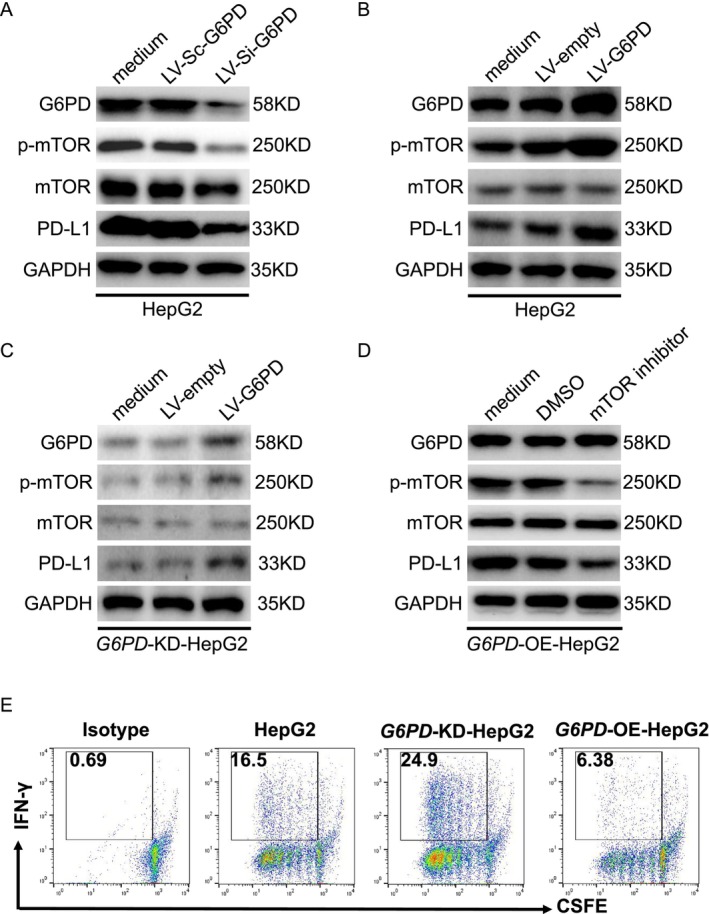
Functional validation of G6PD in HCC. (A‐D) Western blot analysis of G6PD, phosphorylated mTOR (p‐mTOR), mTOR, and PD‐L1 expression in HepG2 cells under different experimental conditions. (A) Knockdown of G6PD with LV‐Si‐G6PD reduces p‐mTOR and PD‐L1 expression compared to the control (LV‐Sc‐G6PD). (B) Overexpression of G6PD (LV‐G6PD) increases p‐mTOR and PD‐L1 expression compared to the control (LV‐empty). (C) Restoration of G6PD expression in G6PD knockdown HepG2 cells (G6PD‐KD‐HepG2) reverses the reduction in p‐mTOR and PD‐L1 expression. (D) Treatment with an mTOR inhibitor decreases p‐mTOR and PD‐L1 expression in G6PD‐overexpressing HepG2 cells (G6PD‐OE‐HepG2), demonstrating the dependency of PD‐L1 regulation on mTOR signaling. (E) Flow cytometry analysis of T cell proliferation shows that G6PD knockdown enhances T cell proliferation (with higher IFN‐γ production), while G6PD overexpression suppresses T cell activation, as indicated by CFSE dilution and IFN‐γ levels.

We further investigated the role of G6PD in regulating PD‐L1 expression in HCC cells. Numerous studies have demonstrated that mTOR is a key upstream regulator of PD‐L1 expression [17, 18, 19], playing a pivotal role in immune evasion by suppressing T cell activity [20]. G6PD exhibited the most significant prognostic value in our survival analysis and Cox regression model, indicating its crucial role in hepatocellular carcinoma (HCC) progression. As a key enzyme in the pentose phosphate pathway, G6PD is strongly implicated in tumor metabolism, oxidative stress regulation, and lactate metabolism. Based on evidence that G6PD regulates immune activation and suppression, we hypothesized that it modulates PD‐L1 expression via the mTOR signaling pathway. To test this hypothesis, we performed Western blot analyses to assess how G6PD knockdown and overexpression influence PD‐L1 and mTOR signaling in HepG2 cells. Results depicted in Figure 9A show that G6PD knockdown significantly reduced phosphorylated mTOR (p‐mTOR) and PD‐L1 levels, suggesting that G6PD modulates PD‐L1 expression via mTOR signaling. Conversely, G6PD overexpression in HepG2 cells increased p‐mTOR and PD‐L1 levels, further supporting that G6PD promotes immune evasion by upregulating PD‐L1 through the mTOR pathway (Figure 9B). Rescue experiments, involving G6PD reintroduction in knockdown cells, restored p‐mTOR and PD‐L1 levels, confirming that G6PD specifically mediates these effects (Figure 9C). Furthermore, treatment with an mTOR inhibitor (Figure 9D) in G6PD‐overexpressing cells significantly reduced p‐mTOR and PD‐L1 levels, reinforcing that G6PD exerts immune‐modulatory effects via mTOR signaling. These findings indicate that G6PD impacts CD8^+^ T cell activation, immune cell infiltration, and PD‐L1 expression via the mTOR pathway, thereby facilitating immune evasion in HCC. By regulating the immune checkpoint PD‐L1, G6PD likely suppresses T cell activity, thus facilitating tumor growth and progression.

### 2.12 Molecular Docking Analysis of G6PD With Candidate Drugs

**FIGURE 10:**
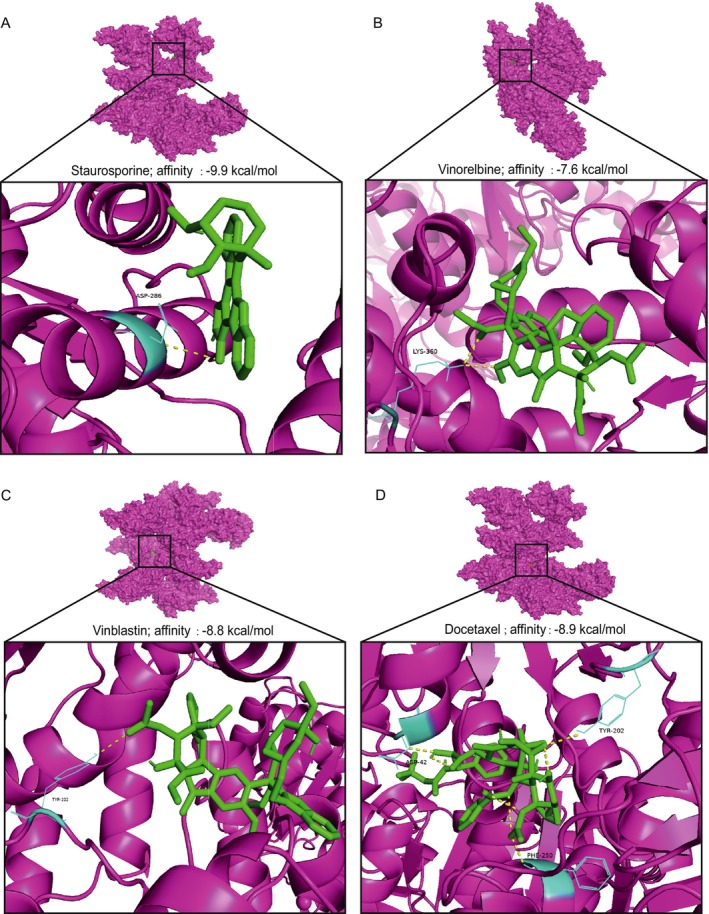
Molecular docking results of G6PD with candidate drugs. (A‐D) Molecular docking of G6PD protein with four selected drugs: (A) Staurosporine_1034, (B) Vinorelbine_2048, (C) Vinblastine_1004, and (D) Docetaxel_1007. The binding interactions are shown in detail, including hydrogen bonds and their respective distances. The overall protein structure is shown in magenta, while the drugs are depicted in green. Close‐up views highlight the binding pockets and specific interactions between G6PD and each drug.

To investigate potential interactions between G6PD and candidate therapeutic compounds, molecular docking was performed using four drugs with the highest predicted sensitivity scores: Staurosporine_1034, Vinorelbine_2048, Vinblastine_1004, and Docetaxel_1007. The docking results revealed strong binding affinities between G6PD and the selected drugs, with binding energies below −7 kcal/mol, suggesting stable interactions. Specifically, Staurosporine_1034 exhibited the highest binding affinity (−9.9 kcal/mol), forming one hydrogen bond within the active site. Vinorelbine_2048 showed a binding affinity of −7.6 kcal/mol, forming two hydrogen bonds that stabilized its interaction with G6PD. Vinblastine_1004 demonstrated a binding affinity of −8.8 kcal/mol, forming a single hydrogen bond. Docetaxel_1007 showed a binding affinity of −8.9 kcal/mol, forming three hydrogen bonds, indicating specific and robust binding. Visualization of the docking results revealed the structural alignment of each drug within the G6PD binding pocket, with key residues interacting through hydrogen bonds and hydrophobic interactions (Figure 10A–D) [21, 22, 23, 24]. These findings suggest that the selected drugs may modulate G6PD activity by directly targeting its active site, potentially affecting lactate metabolism in HCC.

## 3 Discussion

Our study highlights the critical role of lactate metabolism in HCC progression and prognosis, identifying several lactate‐associated genes, particularly G6PD, IK, and CALML5, as significant prognostic markers. These findings support the growing body of evidence indicating that cancer metabolism, particularly lactate production and utilization, significantly impacts tumor behavior and patient outcomes [13, 14, 15].

The association between high lactate production and aggressive tumor features, along with poor prognosis, is well established [25]. Lactate not only supports cancer cell growth and survival but also alters the tumor microenvironment, promoting cancer progression through immunosuppression and angiogenesis [2, 10, 11, 12]. Our results specifically highlight the enzyme G6PD, a key player in the pentose phosphate pathway, involved in reductive biosynthesis and the generation of reducing equivalents. G6PD's role in modulating PD‐L1 expression via the mTOR signaling pathway, as demonstrated in our functional assays, highlights its involvement in immune evasion by HCC cells. This finding establishes a potential link between metabolic reprogramming and immune checkpoint regulation, an important area for therapeutic intervention.

Given the constraints of available resources and experimental feasibility, we focused on validating the most promising therapeutic target first. While IK and CALML5 may also play roles in HCC, their functional significance requires further investigation, which we plan to explore in future studies. Future studies with larger cohorts and functional assays will be necessary to comprehensively elucidate the roles of IK and CALML5 in HCC. The dual role of G6PD in metabolic modulation and immune regulation makes it a promising target for HCC therapy. Drugs that inhibit G6PD activity could not only disrupt cancer cell metabolic homeostasis but also enhance the immune response against tumors. Our molecular docking studies indicate that inhibitors such as staurosporine could disrupt G6PD activity, suggesting a novel approach to curb HCC progression. This therapeutic strategy may be especially effective in patients with high G6PD expression and poor prognosis.

The prognostic model based on lactate‐associated genes effectively stratifies HCC patients into high‐ and low‐risk categories, providing valuable insights for personalized treatment strategies. For example, patients in the high‐risk category may benefit from more aggressive treatment regimens and close monitoring. Furthermore, the model's strong performance across independent datasets highlights its potential utility in clinical settings, assisting in decision‐making and improving patient management.

Despite these promising findings, several challenges persist. The heterogeneity of HCC and the complex interplay of genetic, environmental, and metabolic factors present significant challenges in the uniform application of prognostic models. Future studies should aim to validate these findings in larger, multicenter cohorts and explore the interrelationships among various metabolic pathways in HCC. Additionally, integrating metabolic profiling with immunological and molecular characteristics could generate comprehensive models that capture the multifaceted nature of cancer [26].

Our analysis of immune infiltration emphasizes the intricate relationship between metabolic reprogramming and the immune landscape in HCC. Understanding how metabolic changes affect immune cell function and the efficacy of immunotherapy could lead to the development of combined metabolic and immune‐modulating therapies. For instance, targeting metabolic enzymes alongside immune checkpoint inhibitors may improve therapeutic efficacy against HCC.

In conclusion, this study not only confirms the importance of lactate metabolism in shaping clinical outcomes in HCC patients but also provides a foundation for future research and therapeutic development. G6PD is a key regulator of metabolic reprogramming and immune modulation in HCC. Our bioinformatics and functional validation studies provide strong evidence that G6PD contributes to immune evasion by modulating immune cell activity and immune checkpoint expression, particularly PD‐L1, via the mTOR signaling pathway. These findings suggest that G6PD is not only a potential prognostic biomarker but also a promising target for immune‐based therapies designed to enhance anti‐tumor immunity and overcome immune evasion in HCC.

## 4 Materials and Methods

### 4.1 Data Collection and Preparation

Hepatocellular carcinoma (HCC) data, including RNA‐sequencing and clinical information, were downloaded from The Cancer Genome Atlas (TCGA) portal (https://portal.gdc.cancer.gov/). A total of 374 HCC samples along with clinical data for 371 patients were included. Additionally, 332 lactate‐associated genes were compiled from relevant literature sources, specifically from PMID: 38419085 [16]. An external validation dataset, LIHC‐JP, was also used.

### 4.2 Prognostic Gene Selection and Model Construction

Initially, univariate Cox regression analysis was performed to assess the prognostic value of each lactate‐associated gene. Genes with a *p* < 0.05 were considered significant and were further analyzed using Lasso regression to prevent overfitting and to refine the selection of variables. Multivariate Cox regression analysis was subsequently conducted to finalize the selection of variables, resulting in three lactate‐associated prognostic genes. These genes were used to construct a prognostic model where each patient's risk score was calculated using the formula:

Risk score = ∑(Expression level of gene × coefficient).

The TCGA dataset was divided into training and validation sets, with LIHC‐JP serving as an external validation set. Kaplan–Meier curves were used to analyze survival differences between high‐ and low‐risk groups in these datasets.

### 4.3 Enrichment Analysis

Differentially expressed genes (DEGs) between risk groups were identified based on |log2FC| > 1.0 and a *p* < 0.05. Gene ontology (GO) analysis for biological processes, cellular components, and molecular functions, along with Kyoto Encyclopedia of Genes and Genomes (KEGG) pathway analysis, was conducted to explore the biological significance of these genes. Gene set enrichment analysis (GSEA) was also performed to further understand pathway differences between high‐ and low‐risk groups.

### 4.4 Immune Analysis

The immune and stromal scores were estimated using the ESTIMATE algorithm to assess the impact of the tumor microenvironment on prognosis. Differences in immune cell infiltration between risk groups were analyzed using CIBERSORT. Correlations between gene expression and immune cell levels were assessed using Spearman's rank correlation.

### 4.5 Mutation Analysis

Somatic mutation data from the TCGA dataset were analyzed using the “maftools” R package. Tumor Mutational Burden (TMB) was calculated, and HCC patients were classified into high and low TMB groups based on the median TMB value. Survival differences between these groups were examined.

### 4.6 Drug Sensitivity Analysis

Drug sensitivity was assessed using the “pRRophetic” R package, which predicts IC50 values for chemotherapeutic agents based on gene expression profiles. Drugs showing significant differences in IC50 values between high‐ and low‐risk groups were identified, and their potential efficacy was evaluated.

### 4.7 Single‐Cell RNA Sequencing Analysis

Single‐cell RNA‐seq data from GEO datasets GSE245906 and GSE149614 were processed using the “Seurat” R package. Data quality control, normalization, and batch effect removal were performed, followed by clustering and differential gene expression analysis. The “SingleR” package was used for cell type annotation, and “AUCell” was utilized to quantify lactate‐related gene expression across cell types.

### 4.8 Validation of Gene Expression

The expression levels of key genes, G6PD and IK, were validated using immunohistochemistry data from the Human Protein Atlas (https://www.proteinatlas.org/). This helped confirm their overexpression in HCC tissues compared to normal liver tissue.

### 4.9 Cell Culture and Lentivirus‐Infection

Human normal hepatocytes L02, human hepatocellular carcinoma cells Huh7, HepG2, and MHCC97H were cultured in complete Dulbecco's modified Eagle's medium (DMEM) with 10% fetal bovine serum (FBS) (HyClone Laboratories, Logan, UT, USA) at 37°C in a 5% CO_2_ atmosphere.

To generate stable knockdown or overexpressing cell lines for G6PD, G6PD shRNA plasmids (LV‐Si‐G6PD), G6PD overexpressing plasmids (LV‐G6PD), and empty controls (LV‐Sc‐G6PD/LV‐empty) were purchased from Santa Cruz Biotechnology. After coinfection of shRNA or overexpressing plasmids with the packaging plasmids psPAX2 (Addgene, Cat# 12260) and pMD2.G (Addgene, Cat# 12259) into HEK‐293 T cells, the lentivirus was harvested, titrated, and stored for infecting HepG2 cells. These two cell lines (*G6PD*‐KD‐HepG2 and *G6PD*‐OE‐HepG2) infected with lentivirus were screened with puromycin, and the stable cell lines were further confirmed with detection of WB.

Cells were grown to 50% confluence in six‐well cell culture plates (Nest, China) and transfection was performed using Lipofectamine 2000 (Invitrogen, Cat# 11668030), according to the manufacturer's instructions. In all the co‐transfection experiments, the corresponding empty vectors were used as negative controls to ensure similar DNA concentrations.

### 4.10 Immunohistochemistry (IHC)

The hepatocellular carcinoma (HCC) tissue microarray (TMA) utilized in this study was sourced from Shanghai Outdo Biotechnology (Project No. OD‐CT‐DgLiv03; Ethics Committee Review Opinion No: YB M‐05‐02), comprising 30 paired HCC samples and their corresponding adjacent normal tissues. Immunohistochemical (IHC) staining was conducted on liver tissue sections following standard protocols. Tissue sections were initially heated at 70°C for 30 min in an oven, followed by deparaffinization using xylene and ethanol for 10 min. To quench endogenous peroxidase activity, the sections were incubated in 0.3% hydrogen peroxide (H_2_O_2_) for 20 min. Antigen retrieval was performed with citrate buffer at 95°C for 10 min. The slides were then incubated with primary antibodies against G6PD (1:100 dilution, Cat#AMRe02038, Enkilife) and IK (1:100 dilution, Cat#31833‐1‐AP, Proteintech). Signal detection was achieved using DAKO EnVision horseradish peroxidase (HRP)‐conjugated anti‐rabbit secondary antibody.

### 4.11 Western Blot Analysis

Western blotting was performed to validate the protein expression levels of G6PD and related signaling molecules such as mTOR and PD‐L1. Cells were lysed using RIPA buffer (Cat#P0013D, Beyotime) containing protease and phosphatase inhibitors (Cat#P1045, Beyotime). Protein concentrations were measured using the BCA Protein Assay Kit (Cat#P0009, Beyotime). Equal amounts of protein were separated on SDS‐PAGE gels and transferred to PVDF membranes. The membranes were blocked with 5% nonfat milk and incubated with primary antibodies against G6PD (Cat#sc‐373,886, Santa Cruz Biotechnology, 1:2000 dilution), mTOR (Cat#28273‐1‐AP, Proteintech, 1:1000 dilution), phospho‐mTOR (Cat#67778‐1‐Ig, Proteintech, 1:1000 dilution), PD‐L1 (Cat#83600‐2‐PBS, Proteintech, 1:1000 dilution), and GAPDH(Cat#60004‐1‐Ig, Proteintech, 1:10000 dilution, used as a loading control). Appropriate HRP‐conjugated secondary antibodies (Cat#7074/7076, Cell Signaling Technology, 1:10000 dilution) were used, and protein bands were visualized using enhanced chemiluminescence (ECL) (Cat#A38556, ThermoFisher). Quantification of band intensity was performed using ImageJ software, and relative expression levels were calculated by normalizing to GAPDH.

### 4.12 Flow Cytometry Analysis

Flow cytometry was employed to assess the functional effects of G6PD modulation on T cell proliferation and IFN‐γ secretion. Human CD8^+^ T cells were labeled with CFSE (carboxyfluorescein succinimidyl ester, Cat#C34554, Invitrogen) and stimulated with anti‐CD3 (Cat#341090, Biosciences) and anti‐CD28 (Cat#348047, Biosciences) antibodies. These T cells were cocultured with HepG2 cells, either with knocked down (KD) or overexpressed [27] G6PD, for 3 days. Postincubation, cells were harvested and stained for CD8 (Cat#335805, Biosciences) and IFN‐γ (Cat#562213, Biosciences). Flow cytometric analysis was conducted to determine T cell proliferation (by CFSE dilution) and IFN‐γ production. The proliferation index and the percentage of IFN‐γ+ cells were calculated using FlowJo software.

### 4.13 Structural Analysis of G6PD and Therapeutic Agents

From the “oncoppredict” results, we selected the four drugs with the highest predicted sensitivity for molecular docking analysis. The molecular structures were obtained from PubChem (https://pubchem.ncbi.nlm.nih.gov/), and protein structures were retrieved from the Protein Data Bank (PDB; http://www.rcsb.org/). Protein and ligand files were converted to PDBQT (Protein Data Bank, Partial Charge (Q), and Atom Type (T)) format, water molecules were removed, and polar hydrogens were added to improve docking accuracy. Molecular docking simulations were conducted using AutoDock Vina 1.2.2 (http://autodock.scripps.edu/) to evaluate drug–protein binding interactions. The therapeutic potential of each drug was assessed based on its docking score, which reflects the strength and affinity of the binding [27]. The G6PD and Staurosporine complex structure was visualized by PyMol.

### 4.14 Statistical Analysis

Statistical analyses were performed using R software (version 4.1.2). The Wilcoxon test was used for differential analysis, Spearman's rank correlation for assessing correlations, and the Kaplan–Meier method for survival analysis. The Benjamini‐Hochberg method was applied for multiple‐testing correction. Significant differences were considered at *p* < 0.05.

## Author Contributions

**Hao‐ran Qu:** validation, software, visualization, writing – original draft, writing – review and editing, methodology, supervision. **Chao‐qun Wang:** investigation, data curation, software, validation, visualization. **Su‐juan Sun:** investigation, formal analysis, writing – original draft. **Wen‐wen Zhang:** investigation. **Cheng‐hao Liu:** investigation. **Xuan‐shuang Du:** investigation. **Yao‐yi‐ao Shu:** investigation. **Xi‐cheng Wang:** investigation. **Qin Pan:** writing – review and editing. **Feng‐ling Luo:** writing – review and editing. **Hong‐yan Wu:** funding acquisition, writing – review and editing, resources. **Xiao‐lian Zhang:** resources, project administration, supervision. **Min Liu:** writing – review and editing, funding acquisition, project administration, writing – original draft, supervision, resources, formal analysis, methodology, validation, conceptualization, visualization, investigation, data curation.

## Ethics Statement

Samples were collected with informed consent and in accordance with established biobanking protocols and ethical and legal standards. Tissue samples were approved by the Ethics Review Committee of Shanghai Outdo Biotechnology Co Ltd. (Project No. OD‐CT‐DgLiv03; Ethics Committee Review Opinion No: YB M‐05‐02).

## Conflicts of Interest

The authors declare no conflicts of interest.

## Clinical Trial Registration Number

Not applicable.

## Supporting information


**Data S1.** Supporting Information

## Data Availability

The data that support the findings of this study are available from the corresponding author upon reasonable request.
