# Correction to “Bioinformatics Identification of Lactate‐Associated Genes in Hepatocellular Carcinoma: G6PD's Role in Immune Modulation”

**DOI:** 10.1002/cam4.70870

**Published:** 2025-04-17

Qu HR, Wang CQ, Sun SJ, Zhang WW, Liu CH, Du XS, Shu YY, Wang XC, Pan Q, Luo FL, Wu HY, Zhang XL, Liu M. *Cancer Med*. 2025 Mar;14(6):e70801. doi: 10.1002/cam4.70801.

The first three authors (Qu HR, Wang CQ, Sun SJ) contributed to the work equally and should be regarded as co‐first authors.

We apologize for this error.

