# Supplementary material for: Bioinformatics Identification of Lactate‐Associated Genes in Hepatocellular Carcinoma: G6PD's Role in Immune Modulation
Source: Cancer Med. 2025 Mar 21;14(6):e70801. doi: 10.1002/cam4.70801 (PMC11927016; doi:10.1002/cam4.70801)
Supplement: Supplementary file 1 — Data S1. Supporting Information [file CAM4-14-e70801-s001.docx]

Supplementary Information for

**Bioinformatics Identification of Lactate-Associated Genes in Hepatocellular Carcinoma: G6PD’s Role in Immune Modulation**

Hao-ran Qu^1, 2#^, Chao-qun Wang^3, 4#^, Su-juan Sun^1, 2#^, Wen-wen Zhang^1, 2^, Cheng-hao Liu^1, 2^, Xuan-shuang Du^1, 2^, Yao-yi-ao Shu^3, 4^, Xi-cheng Wang^3, 4^, Qin Pan^1, 2^, Feng-ling Luo^1, 2^, Xiao-lian Zhang^1, 2^*, Min Liu^1, 2^*

**Affiliations**

^1^ State Key Laboratory of Virology, Hubei Province Key Laboratory of Allergy and Immunology, Department of Immunology Wuhan University Taikang Medical School (School of Basic Medical Sciences) Wuhan University, Wuhan, China.

^2^ Frontier Science Center for Immunology and Metabolism, Department of Allergy Zhongnan Hospital, Wuhan University School of Medicine, Wuhan, China.

^3^ Department of Neurosurgery, Renmin Hospital of Wuhan University, Wuhan, China.

^4^ Central Laboratory, Renmin Hospital of Wuhan University, Wuhan, China.

*Corresponding author.

*Prof. Xiao-Lian Zhang, Ph.D., Department of Immunology, Wuhan University Taikang Medical School (School of Basic Medical Sciences), Donghu Road 185#, Wuhan 430071, Hubei Province, P. R. China, E-mail: zhangxiaolian@whu.edu.cn

*Assoc. Prof. Min Liu, Ph.D., Department of Immunology, Wuhan University Taikang Medical School (School of Basic Medical Sciences), Donghu Road 185#, Wuhan 430071, Hubei Province, P. R. China, E-mail: [mliu@whu.edu.cn](mailto:mliu@whu.edu.cn)

**The PDF file includes:**

Supplementary Figure S1-S3


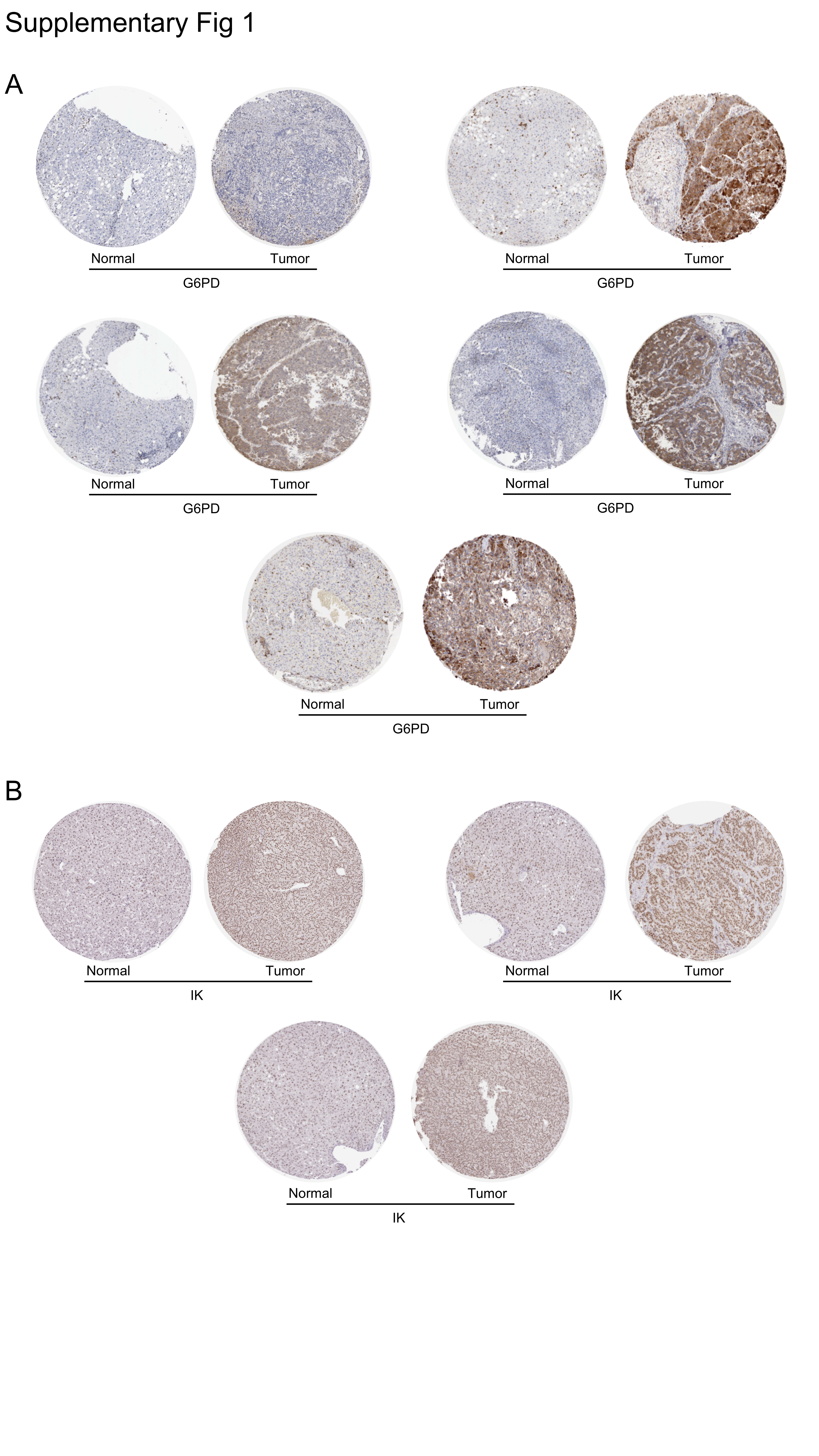


**Supplementary Figure 1: G6PD and IK expression in normal and tumor liver tissues by IHC. (A, B)** Immunohistochemical (IHC) staining of normal and tumor liver tissues for G6PD and IK, were validated using IHC data from the Human Protein Atlas (https://www.proteinatlas.org/).


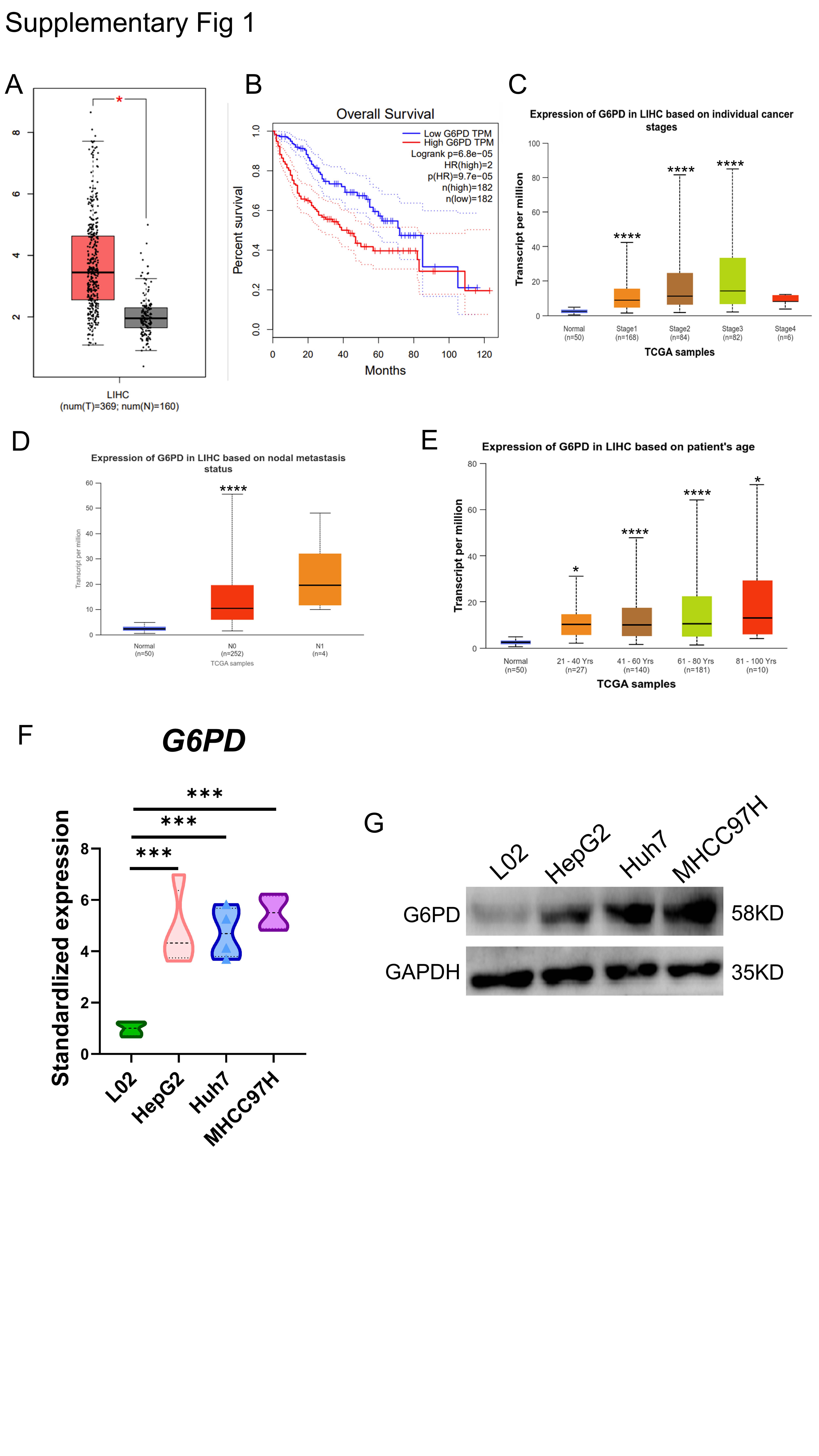


**Supplementary Figure 2: G6PD expression and its clinical relevance in LIHC.** (A) Comparison of G6PD mRNA expression levels between normal and tumor tissues in the LIHC dataset from TCGA reveals significantly higher expression in tumor samples compared to normal tissues. (B) Kaplan-Meier survival analysis for overall survival (OS) in LIHC patients stratified by high and low G6PD expression levels, showing that high G6PD expression is associated with poor OS. (C) G6PD expression across different cancer stages in the LIHC dataset shows an increase with advancing cancer stage. (D) G6PD expression based on nodal metastasis status shows significantly higher levels in tumors with lymph node metastasis (N1) compared to non-metastatic (N0) samples. (E) G6PD expression in LIHC patients stratified by age groups shows significant differences, with higher levels observed in older patients. (F) G6PD expression across liver cancer cell lines (HepG2, Huh7, MHCC97H) and the normal liver cell line (L02) shows significantly higher expression in cancer cell lines. (G) Western blot analysis of G6PD protein expression in liver cancer cell lines (HepG2, Huh7, HCC97H) and the normal liver cell line (L02) shows protein levels consistent with mRNA expression, confirming upregulation in cancer cell lines.


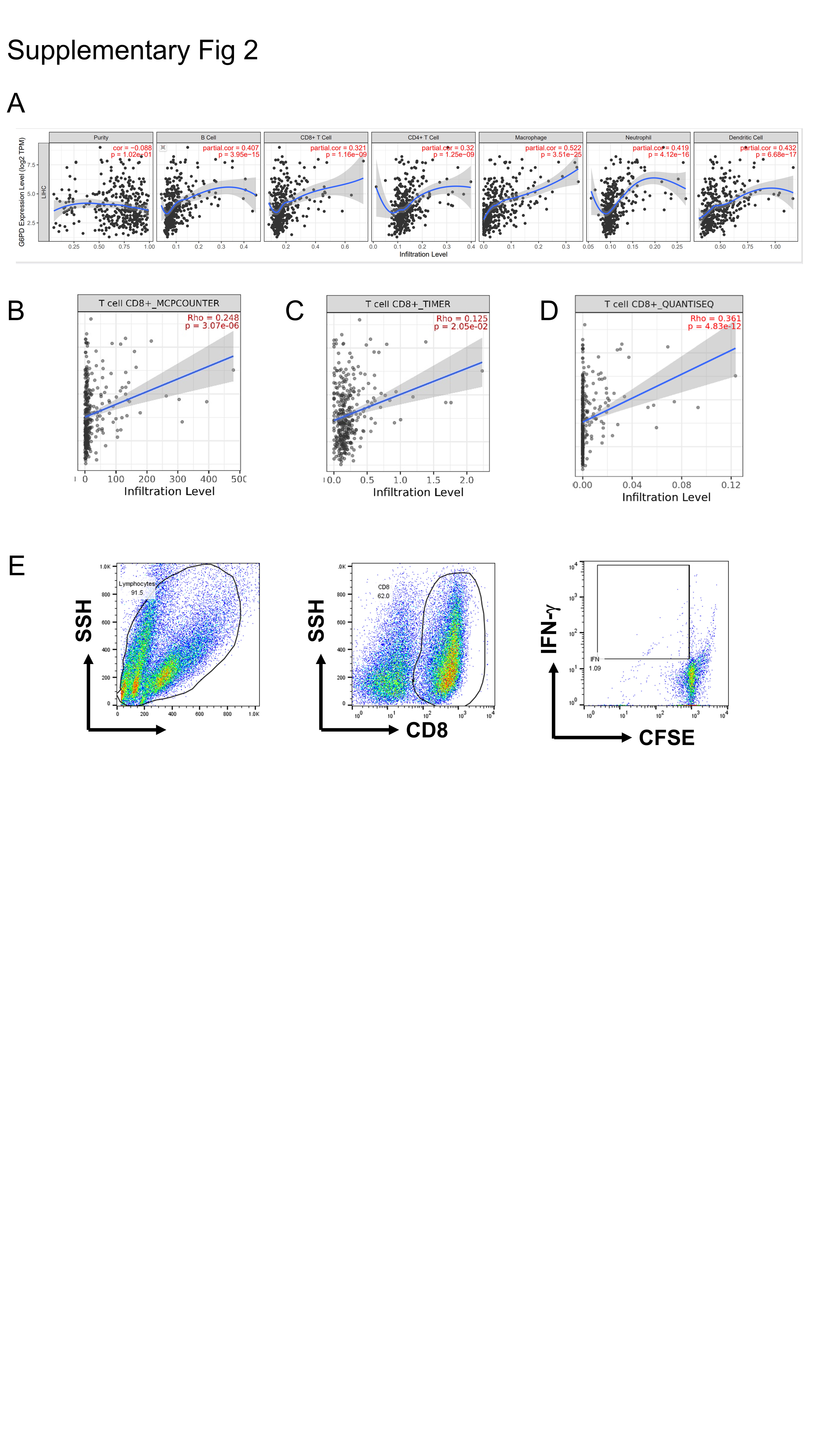


**Supplementary Figure 3: Correlation of G6PD Expression with Immune Infiltration and CD8^+^ T Cell Functionality in LIHC.** (A) Spearman correlation analysis between G6PD expression and infiltration levels of various immune cell types, including B cells, CD8^+^ T cells, CD4^+^ T cells, macrophages, neutrophils, and dendritic cells, from the TIMER database shows that G6PD expression is significantly positively correlated with CD8^+^ T cell and macrophage infiltration levels. (B-D) Scatter plots showing the correlation between G6PD expression and CD8^+^ T cell infiltration, assessed by the MCPcounter, TIMER, and QUANTISEQ algorithms, respectively, confirm a significant positive correlation in all three methods. (E) Flow cytometry analysis assessing the impact of G6PD modulation on CD8^+^ T cell proliferation and IFN-γ secretion. Human CD8^+^ T cells were labeled with CFSE and co-cultured with HepG2 cells with G6PD knockdown (KD) or overexpression (OE). After 3 days, cells were harvested, stained for CD8 and IFN-γ, and analyzed by flow cytometry to assess T cell proliferation (by CFSE dilution) and IFN-γ production.
